# Supplementary material for: Copper acquisition is essential for plant colonization and virulence in a root-infecting vascular wilt fungus
Source: PLoS Pathog. 2024 Nov 4;20(11):e1012671. doi: 10.1371/journal.ppat.1012671 (PMC11563359; doi:10.1371/journal.ppat.1012671)
Supplement: S1 Fig — (A) Schematic diagram of the domain structure of F. oxysporum Mac1. The copper fist DNA-binding domain containing the conserved RGHR and a GRP residues and the copper-binding domain containing two cysteine-rich motifs are located at the N- and C-terminus, respectively. (B) Alignment of Mac1 homologs from different fungi. Identical amino acid residues are highlighted. (C) Phylogenetic tree of Mac1 homologs from different fungi generated using the Maximum Likelihood method and JTT matrix-based model. Bootstrap consensus tree inferred from 1000 replicates. The percentage of replicate trees in which the associated taxa clustered together in the bootstrap test are shown at the base of each clade. The transcription factor Pro1 from F. oxysporum was used as outgroup for the analysis. Protein sequences were aligned with MAFFT and evolutionary analyses were conducted in MEGA. (PDF) [file ppat.1012671.s001.pdf]

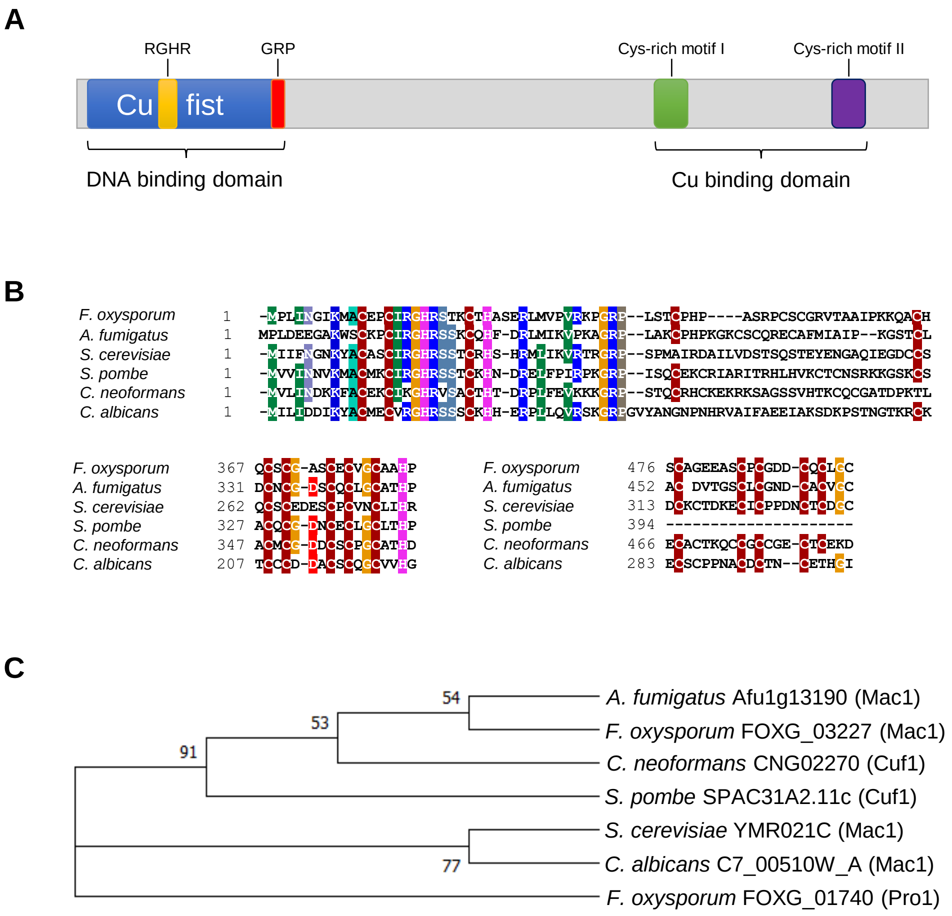

S1 Fig. *Fusarium oxysporum* Mac1. (A) Schematic diagram of the domain structure of *F. oxysporum* Mac1. The copper fist DNA-binding domain containing the conserved RGHR and a GRP residues and the copper-binding domain containing two cysteine-rich motifs are located at the N- and C-terminus, respectively. **(B)** Alignment of Mac1 homologs from different fungi. Identical amino acid residues are highlighted. **(C)** Phylogenetic tree of Mac1 homologs from different fungi generated using the Maximum Likelihood method and JTT matrix-based model. Bootstrap consensus tree inferred from 1000 replicates. The percentage of replicate trees in which the associated taxa clustered together in the bootstrap test are shown at the base of each clade. The transcription factor Pro1 from *F. oxysporum* was used as outgroup for the analysis. Protein sequences were aligned with MAFFT and evolutionary analyses were conducted in MEGA.
