# Supplementary material for: Copper acquisition is essential for plant colonization and virulence in a root-infecting vascular wilt fungus
Source: PLoS Pathog. 2024 Nov 4;20(11):e1012671. doi: 10.1371/journal.ppat.1012671 (PMC11563359; doi:10.1371/journal.ppat.1012671)
Supplement: S2 Fig — (A) Physical map of the F. oxysporum mac1 locus in the wt and the mac1Δ strains. Relative positions of restriction sites, PCR primers and the probe used in the Southern blot analysis are indicated. HygR, hygromycin resistance gene. (B) Southern blot analysis of putative mac1Δ deletion mutants. Genomic DNA of the wt strain and twelve independent hygromycin resistant transformants was treated with BamHI, separated on a 0.7% agarose gel, transferred to a nylon membrane, and hybridized with the DNA probe corresponding to the 5’ flanking region of mac1 indicated in (A). Molecular sizes of the hybridizing bands are indicated on the left. Transformants #1, #7, #10 and #11 show hybridizing bands consistent with homologous replacement of the mac1 gene with the hygromycin resistance cassette. (C) Physical map of the F. oxysporum mac1 locus in the mac1Δ mutant and the complemented mac1Stag strain. Relative positions of PCR primers are indicated. (D) Agarose gel electrophoresis of PCR products obtained using the primer pair Mac1-5’-F and Mac1-Stag-R with genomic DNA extracted from the indicated strains. M, molecular size markers.The presence of the 2916 bp amplification band is consistent with insertion of the mac1Stag allele at the native mac1 locus. (PDF) [file ppat.1012671.s002.pdf]

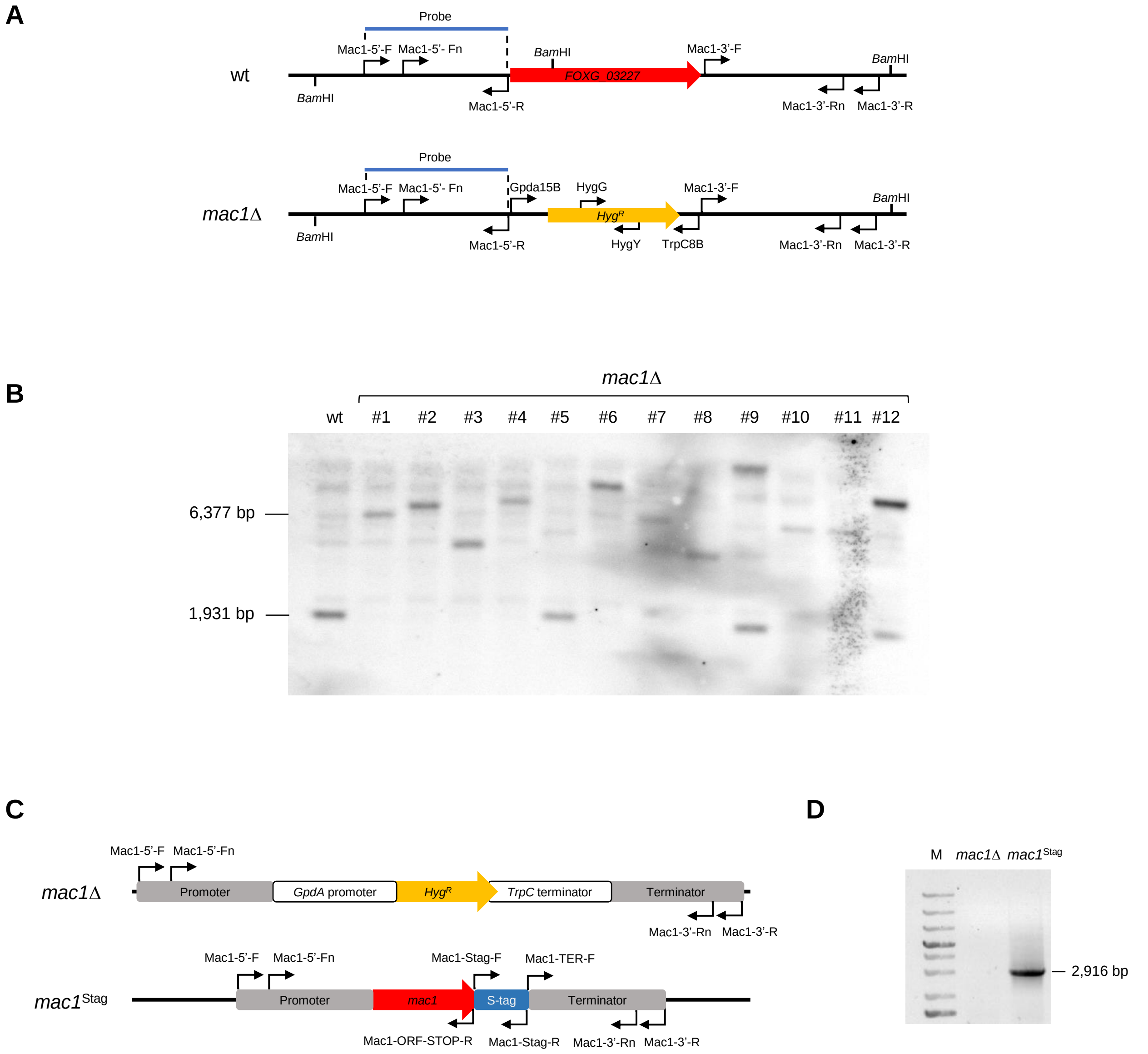

S2 Fig. Generation of *mac1* knockout and complemented strains. (A) Physical map of the *F. oxysporum mac1* locus in the wt and the *mac1*Δ strains. Relative positions of restriction sites, PCR primers and the probe used in the Southern blot analysis are indicated. *Hyg*^{R}, hygromycin resistance gene. **(B)** Southern blot analysis of putative *mac1*Δ deletion mutants. Genomic DNA of the wt strain and twelve independent hygromycin resistant transformants was treated with *Bam*HI, separated on a 0.7% agarose gel, transferred to a nylon membrane, and hybridized with the DNA probe corresponding to the 5' flanking region of *mac1* indicated in **(A)**. Molecular sizes of the hybridizing bands are indicated on the left. Transformants #1, #7, #10 and #11 show hybridizing bands consistent with homologous replacement of the *mac1* gene with the hygromycin resistance cassette. **(C)** Physical map of the *F. oxysporum mac1* locus in the *mac1*Δ mutant and the complemented *mac1*^{Stag} strain. Relative positions of PCR primers are indicated. **(D)** Agarose gel electrophoresis of PCR products obtained using the primer pair Mac1-5'-F and Mac1-Stag-R with genomic DNA extracted from the indicated strains. M, molecular size markers.The presence of the 2916 bp amplification band is consistent with insertion of the *mac1*^{Stag} allele at the native *mac1* locus.
