# Supplementary material for: Copper acquisition is essential for plant colonization and virulence in a root-infecting vascular wilt fungus
Source: PLoS Pathog. 2024 Nov 4;20(11):e1012671. doi: 10.1371/journal.ppat.1012671 (PMC11563359; doi:10.1371/journal.ppat.1012671)
Supplement: S3 Fig — Alignment of the F. oxysporum Ctr1a and Ctr1b amino acid sequences with Ctr1 homologs from other fungi. Ctr1 regions absent in F. oxysporum Ctr1b are indicated with red boxes. (PDF) [file ppat.1012671.s003.pdf]

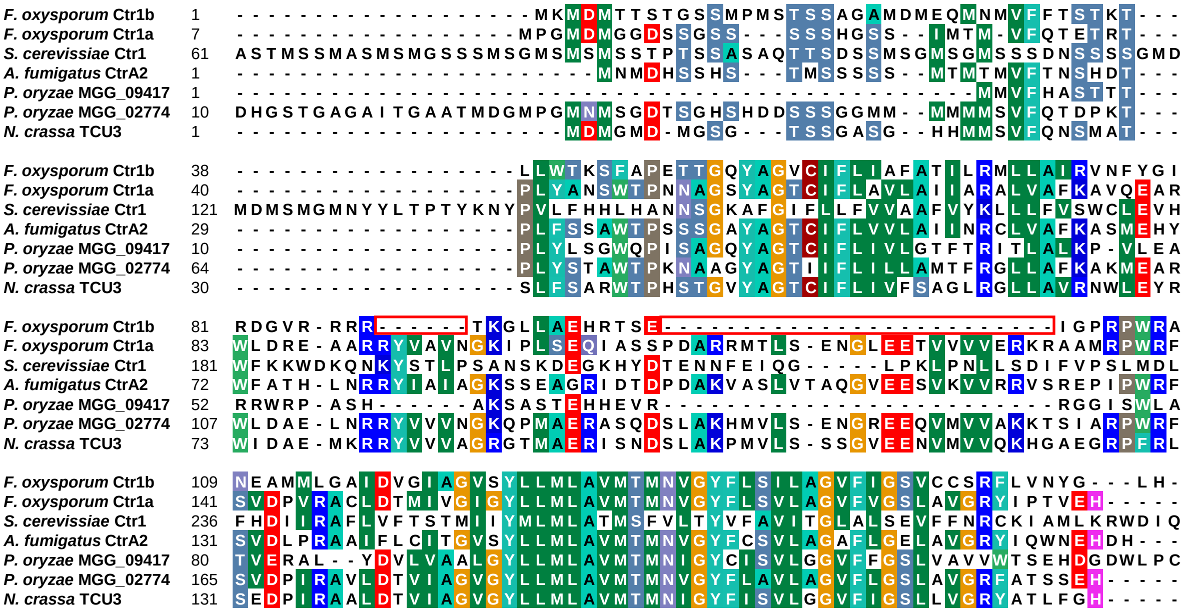


**S3 Fig. Alignment of the two *F. oxysporum* isoforms of the high affinity copper transporter Ctr1.** Alignment of the *F. oxysporum* Ctr1a and Ctr1b amino acid sequences with Ctr1 homologs from other fungi. Ctr1 regions absent in *F. oxysporum* Ctr1b are indicated with red boxes.
