# Supplementary material for: Copper acquisition is essential for plant colonization and virulence in a root-infecting vascular wilt fungus
Source: PLoS Pathog. 2024 Nov 4;20(11):e1012671. doi: 10.1371/journal.ppat.1012671 (PMC11563359; doi:10.1371/journal.ppat.1012671)
Supplement: S4 Fig — (A, B) Phylogenetic trees of Ctr and Fre homologs from different fungi generated using the Maximum Likelihood method and JTT matrix-based model. Bootstrap consensus tree inferred from 1000 replicates. The percentage of replicate trees in which the associated taxa clustered together in the bootstrap test are shown at the base of each clade. The Cu2+-transporting P-type ATPase Ccc2 (A) and the soluble fumarate reductase FRD1 (B) from S. cerevisiae were used as outgroups for the analysis. Protein sequences were aligned with MAFFT and evolutionary analyses were conducted in MEGA. Proteins encoded by F. oxysporum genes induced under copper limiting conditions are in bold. (PDF) [file ppat.1012671.s004.pdf]

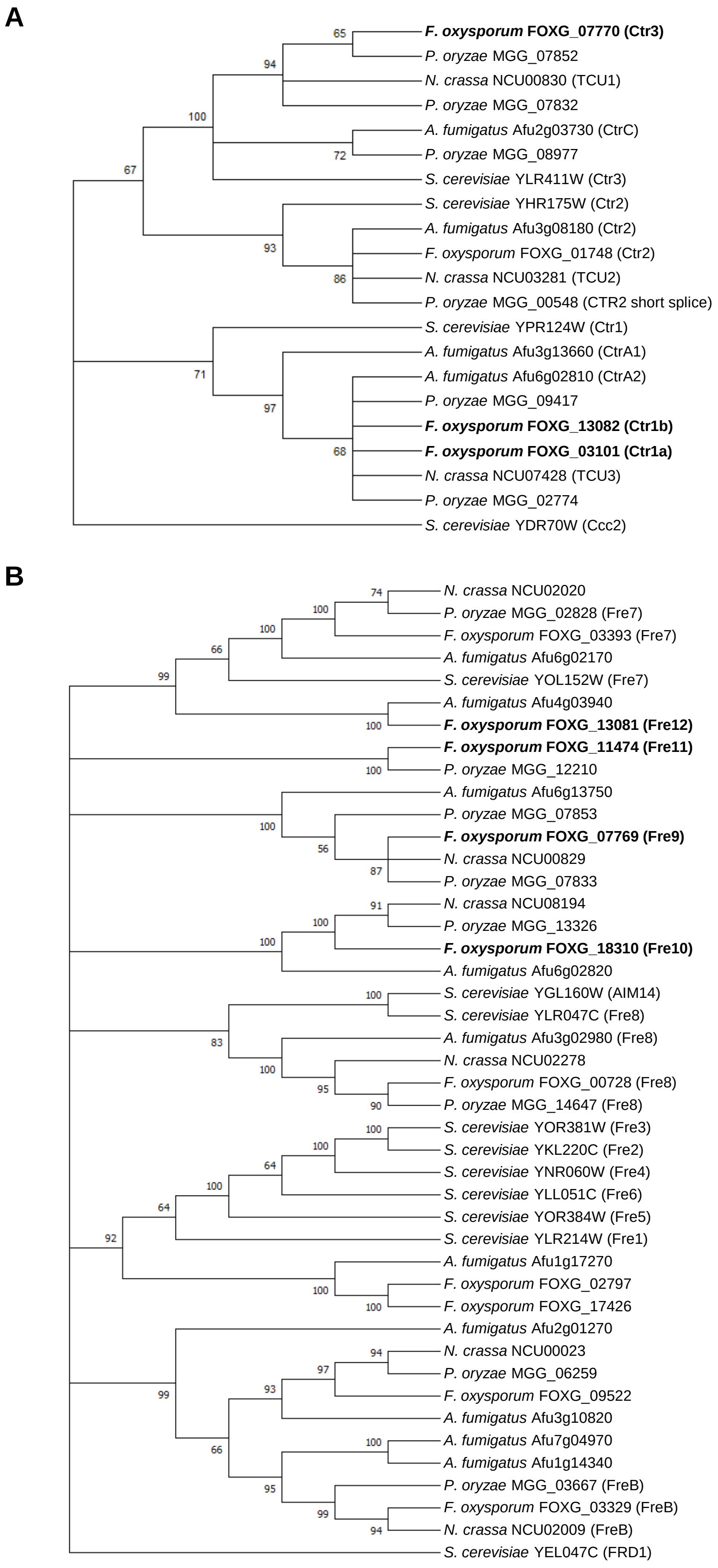

S4 Fig. Phylogenetic analysis of Ctr high affinity copper transporters and Fre metalloreductases in fungi. (A, B) Phylogenetic trees of Ctr and Fre homologs from different fungi generated using the Maximum Likelihood method and JTT matrix-based model. Bootstrap consensus tree inferred from 1000 replicates. The percentage of replicate trees in which the associated taxa clustered together in the bootstrap test are shown at the base of each clade. The Cu^{2+}-transporting P-type ATPase Ccc2 **(A)** and the soluble fumarate reductase FRD1 **(B)** from *S. cerevisiae* were used as outgroups for the analysis. Protein sequences were aligned with MAFFT and evolutionary analyses were conducted in MEGA. Proteins encoded by *F. oxysporum* genes induced under copper limiting conditions are in bold.
