# Supplementary material for: Copper acquisition is essential for plant colonization and virulence in a root-infecting vascular wilt fungus
Source: PLoS Pathog. 2024 Nov 4;20(11):e1012671. doi: 10.1371/journal.ppat.1012671 (PMC11563359; doi:10.1371/journal.ppat.1012671)
Supplement: S5 Fig — (A) Putative consensus DNA sequence bound by F. oxysporum Mac1, identified by exporting the genomic sequences around the peak locations (500 bp) of Mac1 binding sites obtained by ChIP-seq and submitting them to BLASTn analysis. Within these regions the consensus sequence TGCTCA was identified. (B-G). Abundance of RNA-seq transcript reads of the wt (dark blue) or the mac1Δ strain (red) under -Cu conditions (RNA-seq, upper graphs); or of gDNA reads from ChIP-seq analysis in the mac1Stag strain under -Cu (grey) or +Cu (light blue) conditions (ChIP-seq, lower graphs). Data are represented as base-level coverage to three Fol4287 gene clusters (B-D) or genes (E-G). Genes are indicated as red boxes and putative Mac1 binding sites on each strand by black triangles. (PDF) [file ppat.1012671.s005.pdf]

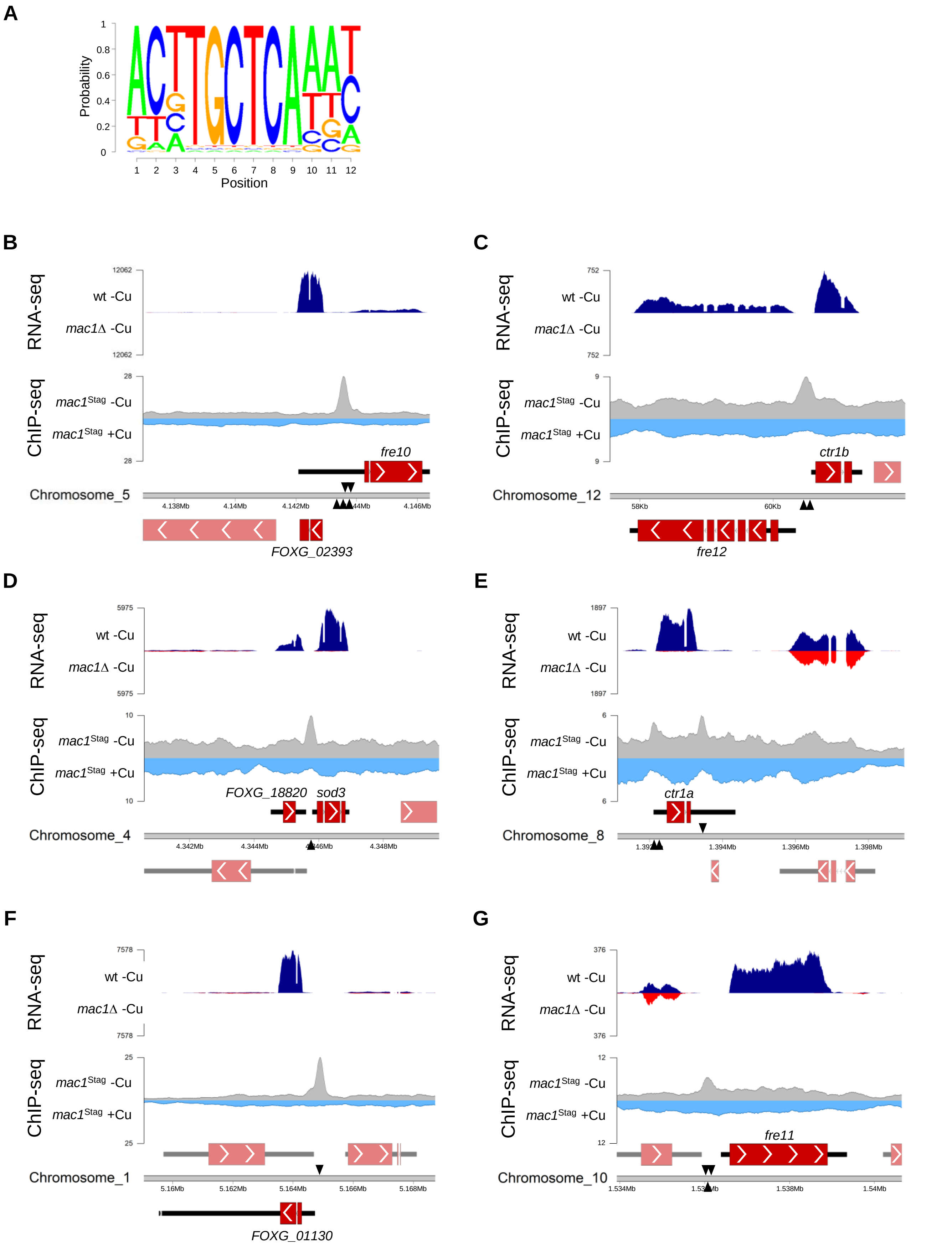

S5 Fig. *F. oxysporum* Mac1 directly activates transcription of copper deficiency response genes. (A) Putative consensus DNA sequence bound by *F. oxysporum* Mac1, identified by exporting the genomic sequences around the peak locations (500 bp) of Mac1 binding sites obtained by ChIP-seq and submitting them to BLASTn analysis. Within these regions the consensus sequence TGCTCA was identified. **(B-G).** Abundance of RNA-seq transcript reads of the wt (dark blue) or the *mac1Δ* strain (red) under -Cu conditions (RNA-seq, upper graphs); or of gDNA reads from ChIP-seq analysis in the *mac1*^{Stag} strain under -Cu (grey) or +Cu (light blue) conditions (ChIP-seq, lower graphs). Data are represented as base-level coverage to three *Fol4287* gene clusters **(B-D)** or genes **(E-G)**. Genes are indicated as red boxes and putative Mac1 binding sites on each strand by black triangles.
