# Supplementary material for: Copper acquisition is essential for plant colonization and virulence in a root-infecting vascular wilt fungus
Source: PLoS Pathog. 2024 Nov 4;20(11):e1012671. doi: 10.1371/journal.ppat.1012671 (PMC11563359; doi:10.1371/journal.ppat.1012671)
Supplement: S6 Fig — (A) Physical map of the F. oxysporum mac1clover DNA construct. Relative positions of PCR primers are indicated. (B) Agarose gel electrophoresis of PCR products obtained using primer pairs Mac1-qPCR-F and EYFPrev with genomic DNA extracted from the indicated transformants. M, molecular size markers. (C) Colony phenotypes of the indicated strains grown for 2 d at 28°C on MM+TE-Cu (20 mM NaNO3) without any copper supply. Scale bar, 0.5 cm. (D) Transcript levels of the indicated genes in the indicated strains transferred for 6 h to MM-TE-Cu with (+Cu) or without (-Cu) 100 μM CuSO4 were measured by real-time RT-qPCR and expressed relative to those of the wt strain in -Cu. Bars represent standard deviations (n = 3, biological replicates). p-vaules: ns>0.05, *<0.05, **<0.01 versus -Cu, in each strain, according to two-tailed unpaired Student’s t test. (PDF) [file ppat.1012671.s006.pdf]

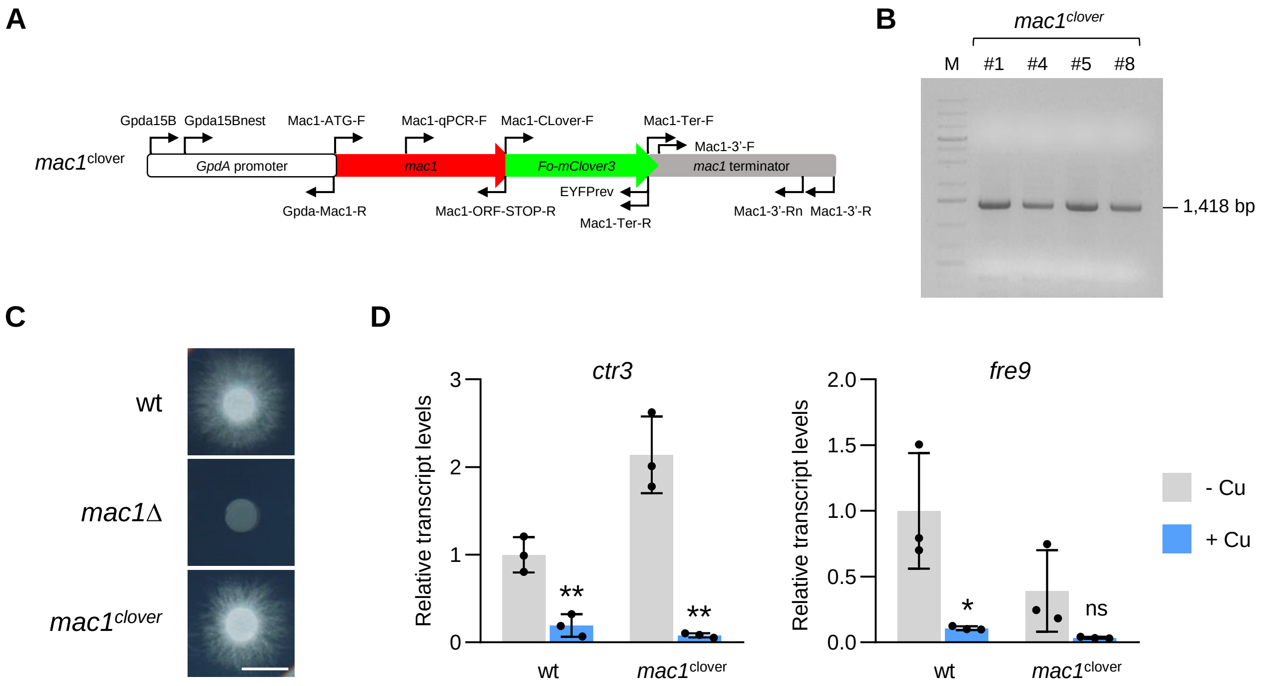

S6 Fig. Generation of a *Fusarium oxysporum mac1*clover strain. (A) Physical map of the *F. oxysporum mac1*clover DNA construct. Relative positions of PCR primers are indicated. **(B)** Agarose gel electrophoresis of PCR products obtained using primer pairs Mac1-qPCR-F and EYFPrev with genomic DNA extracted from the indicated transformants. M, molecular size markers. **(C)** Colony phenotypes of the indicated strains grown for 2 d at 28 ºC on MM+TE^{-Cu} (20 mM $NaNO_3$) without any copper supply. Scale bar, 0.5 cm. **(D)** Transcript levels of the indicated genes in the indicated strains transferred for 6 h to MM-TE^{-Cu} with (+Cu) or without (-Cu) 100 µM $CuSO_4$ were measured by real-time RT-qPCR and expressed relative to those of the wt strain in -Cu. Bars represent standard deviations (*n* = 3, biological replicates). *p*-vaules: ns>0.05, *<0.05, **<0.01 versus -Cu, in each strain, according to two-tailed unpaired Student's *t* test.
