# Supplementary material for: Copper acquisition is essential for plant colonization and virulence in a root-infecting vascular wilt fungus
Source: PLoS Pathog. 2024 Nov 4;20(11):e1012671. doi: 10.1371/journal.ppat.1012671 (PMC11563359; doi:10.1371/journal.ppat.1012671)
Supplement: S7 Fig — (A) Comparative analysis of differentially expressed genes from RNA-seq analysis of the wt strain in axenic culture in MM+TE-Cu (-Cu) or during infection of tomato plants at 2 or 6 dpi. Upregulated (yellow) or downregulated (purple) sets of genes showing significant differential expression in the indicated condition versus axenic culture under copper sufficiency (MM+TE-Cu + 100 μM CuSO4). Vertical bars show the intersection between sets and numbers refer to genes unique for each intersection. Genes upregulated both in copper limitation and under infection conditions are highlighted in red with gene names indicated above. Data were visualized using ComplexUpSet. (B) The indicated strains were spot-inoculated on top of a cellophane membrane on PDA plates with (+Cu) or without 5 μM CuSO4, grown for 3 d at 28°C and imaged (Before). The cellophane with the fungal colony was removed and plates were incubated for an additional day to determine the presence of mycelial growth on the plate, indicating penetration of the cellophane (After). Scale bar, 2 cm. (PDF) [file ppat.1012671.s007.pdf]

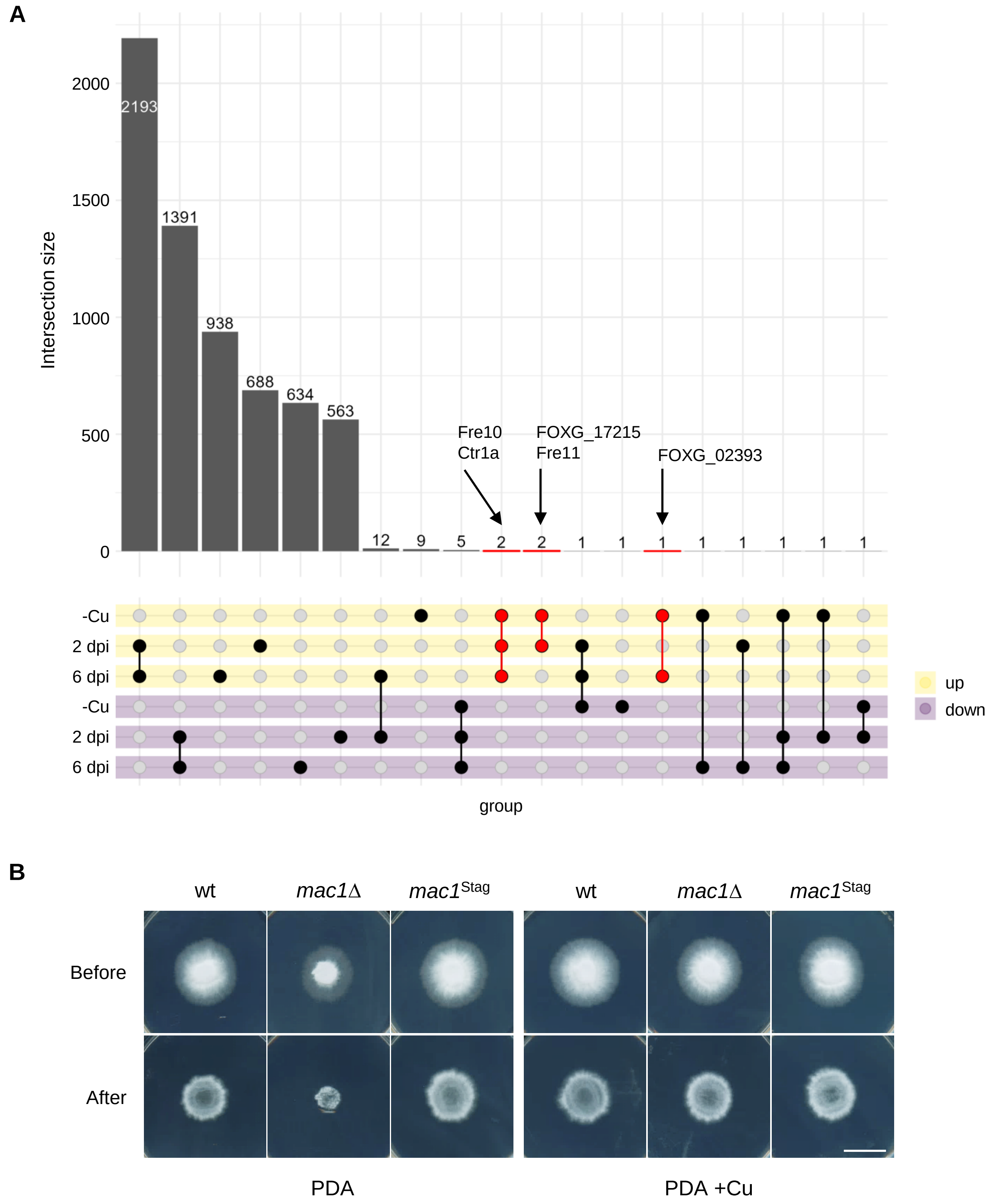

S7 Fig. Comparative transcriptional analysis in -Cu and plant infection versus +Cu conditions and invasive growth assay on cellophane-covered plates. (A) Comparative analysis of differentially expressed genes from RNA-seq analysis of the wt strain in axenic culture in MM+TE^{-Cu} (-Cu) or during infection of tomato plants at 2 or 6 dpi. Upregulated (yellow) or downregulated (purple) sets of genes showing significant differential expression in the indicated condition versus axenic culture under copper sufficiency (MM+TE^{-Cu} + 100 µM $CuSO_4$). Vertical bars show the intersection between sets and numbers refer to genes unique for each intersection. Genes upregulated both in copper limitation and under infection conditions are highlighted in red with gene names indicated above. Data were visualized using ComplexUpSet. **(B)** The indicated strains were spot-inoculated on top of a cellophane membrane on PDA plates with (+Cu) or without 5 µM $CuSO_4$, grown for 3 d at 28 ºC and imaged (Before). The cellophane with the fungal colony was removed and plates were incubated for an additional day to determine the presence of mycelial growth on the plate, indicating penetration of the cellophane (After). Scale bar, 2 cm.
