# Supplementary material for: Copper acquisition is essential for plant colonization and virulence in a root-infecting vascular wilt fungus
Source: PLoS Pathog. 2024 Nov 4;20(11):e1012671. doi: 10.1371/journal.ppat.1012671 (PMC11563359; doi:10.1371/journal.ppat.1012671)
Supplement: S8 Fig — (A, C) Physical maps of the F. oxysporum ctr3 and ctr1a loci in the wt and in the ctr3Δ (A) and ctr1aΔ (C) strains, respectively. Relative positions of restriction sites, PCR primers and the probes used in the Southern blots are indicated. HygR, hygromycin resistance gene. NeoR, neomycin resistance gene. (B, D, E) Southern blot analysis of putative ctr3Δ (B), ctr1aΔ (D) and ctr3Δctr1aΔ (E) deletion mutants. Genomic DNA of the wt strain and independent hygromycin or neomycin resistant transformants was treated with EcoRI (B) or BamHI (D, E), separated on 0.7% agarose gels, transferred to nylon membranes and hybridized with a DNA probe corresponding to the 5’ flanking region of ctr3 (B, indicated in A) or to the 3’ flanking region of ctr1a (D, E indicated in C). Molecular sizes of the hybridizing bands are indicated on the left. (F) Colony phenotypes of the indicated strains after 2 d growth on MM+TE-Cu, supplemented with the indicated concentrations of CuSO4. Scale bar, 1 cm. The colonies of wt and mac1Δ are the same as those shown in Fig 1A and are repeated here for clarity. Scale bar, 1 cm. (G) Kaplan-Meier plot showing survival of groups of 10 tomato plants (cv. Momotaro) inoculated by dipping the roots into a suspension of 5x10^6^ microconidia/ml of the indicated fungal strains or water (Mock). Data shown are from one representative experiment. Experiments were performed at least two times with similar results. p-value: ***<0.001 versus the wt according to Log-rank (Mantel-Cox) test. (PDF) [file ppat.1012671.s008.pdf]

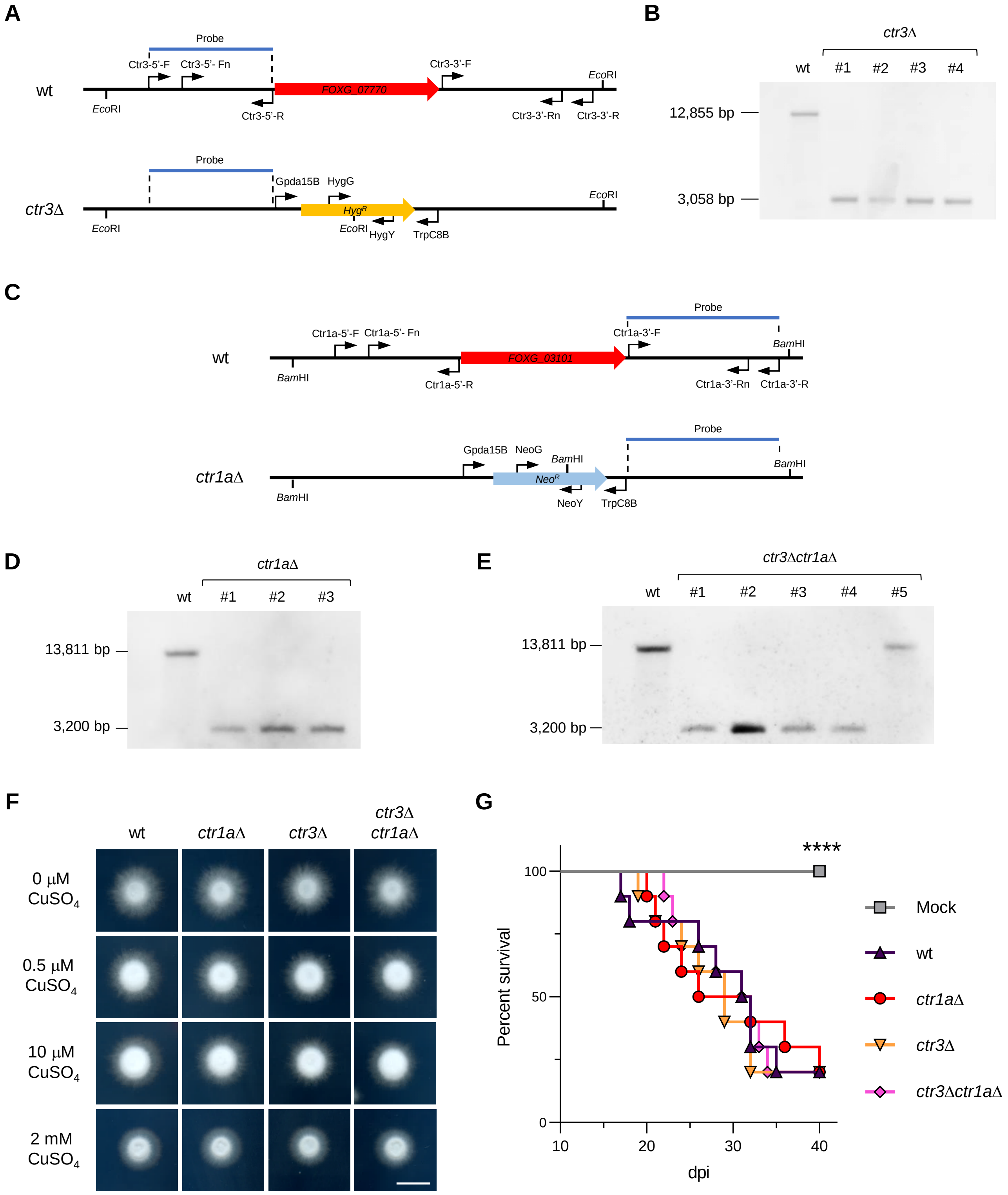

S8 Fig. Targeted deletion of the high-affinity copper transporters Ctr3 and Ctr1a in *F. oxysporum*. (A, C) Physical maps of the *F. oxysporum ctr3* and *ctr1a* loci in the wt and in the *ctr3Δ* **(A)** and *ctr1aΔ* **(C)** strains, respectively. Relative positions of restriction sites, PCR primers and the probes used in the Southern blots are indicated. Hyg^R, hygromycin resistance gene. Neo^R, neomycin resistance gene. **(B, D, E)** Southern blot analysis of putative *ctr3Δ* **(B)**, *ctr1aΔ* **(D)** and *ctr3Δctr1aΔ* **(E)** deletion mutants. Genomic DNA of the wt strain and independent hygromycin or neomycin resistant transformants was treated with *Eco*RI **(B)** or *Bam*HI **(D, E)**, separated on 0.7% agarose gels, transferred to nylon membranes and hybridized with a DNA probe corresponding to the 5' flanking region of *ctr3* **(B, indicated in A)** or to the 3' flanking region of *ctr1a* **(D, E indicated in C)**. Molecular sizes of the hybridizing bands are indicated on the left. **(F)** Colony phenotypes of the indicated strains after 2 d growth on MM+TE^{-Cu}, supplemented with the indicated concentrations of $CuSO_4$. Scale bar, 1 cm. The colonies of wt and *mac1Δ* are the same as those shown in Fig 1A and are repeated here for clarity. Scale bar, 1 cm. **(G)** Kaplan-Meier plot showing survival of groups of 10 tomato plants (cv. Momotaro) inoculated by dipping the roots into a suspension of $5x10^6$ microconidia/ml of the indicated fungal strains or water (Mock). Data shown are from one representative experiment. Experiments were performed at least two times with similar results. *p*-value: ***<0.001 versus the wt according to Log-rank (Mantel-Cox) test.
