# Supplementary material for: Copper acquisition is essential for plant colonization and virulence in a root-infecting vascular wilt fungus
Source: PLoS Pathog. 2024 Nov 4;20(11):e1012671. doi: 10.1371/journal.ppat.1012671 (PMC11563359; doi:10.1371/journal.ppat.1012671)
Supplement: S9 Fig — (A) Physical maps of the DNA constructs used for over-expression of ctr3 and fre9 in mac1Δ. Relative positions of PCR primers are indicated. (B, C) Agarose gel electrophoresis of PCR products obtained using primer pairs Gpda4 and Ctr3-FOXG_07770-R (B), and Gpda4 and Fre9-3’-Rn (C) using genomic DNA extracted from the indicated nourseothricin-resistant transformants or from mac1Δ as a negative control. M, molecular size markers. (D, E) Real-time RT-qPCR analysis was performed in the indicated strains grown in the absence (-Cu) or presence (+Cu) of 100 μM CuSO4. Transcript levels of the indicated genes are expressed relative to those of the wt strain in -Cu conditions. Bars represent standard deviations (n = 3, biological replicates). p-values: ns>0.05, *<0.05, **<0.01 versus mac1Δ, under the same condition, according to two-tailed unpaired Student’s t test. (PDF) [file ppat.1012671.s009.pdf]

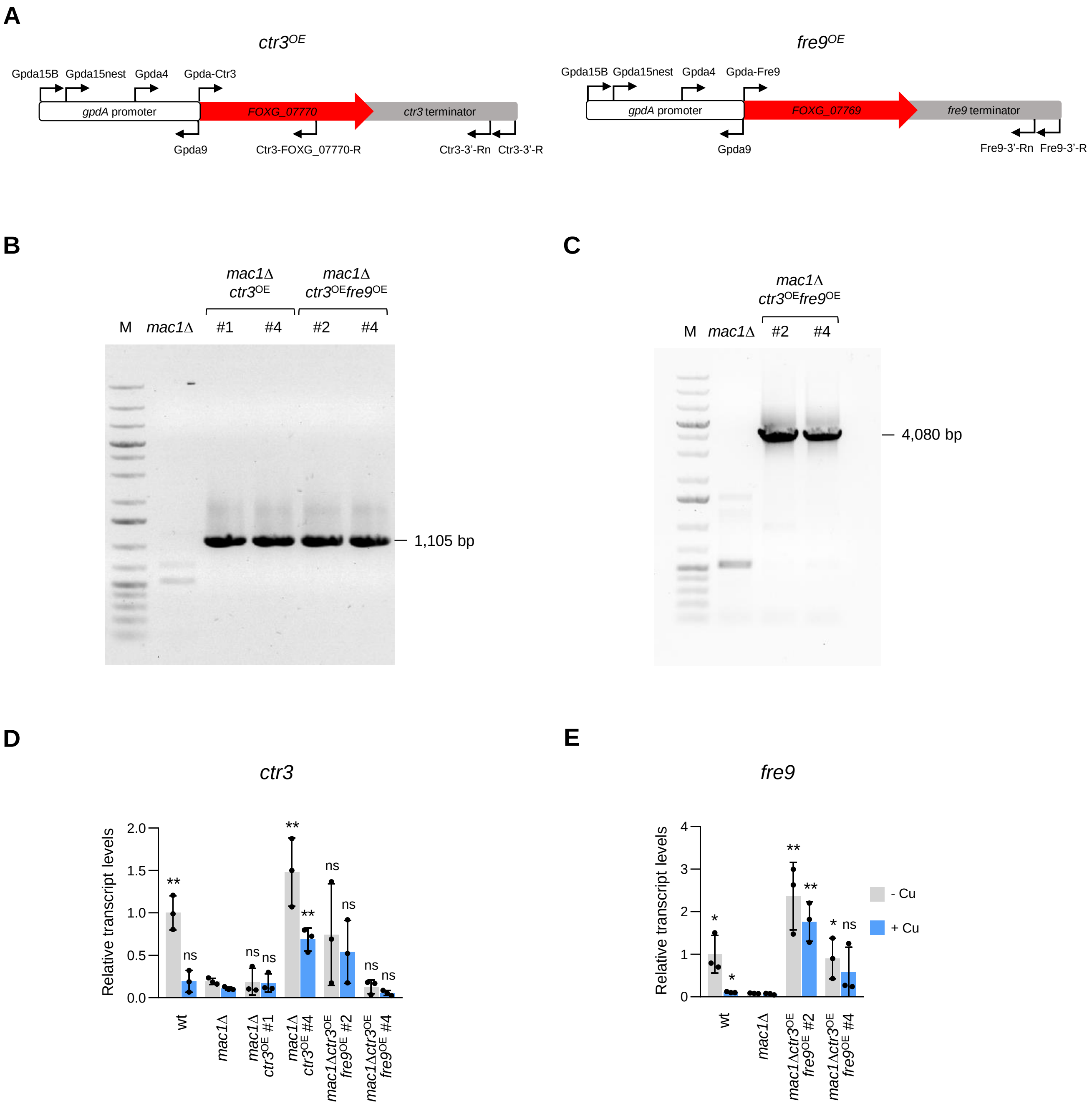

S9 Fig. Generation of *F. oxysporum* strains overexpressing *ctr3* and/or *fre9* in a *mac1*Δ background. (A) Physical maps of the DNA constructs used for over-expression of *ctr3* and *fre9* in *mac1*Δ. Relative positions of PCR primers are indicated. **(B, C)** Agarose gel electrophoresis of PCR products obtained using primer pairs Gpda4 and Ctr3-FOXG_07770-R **(B)**, and Gpda4 and Fre9-3'-Rn **(C)** using genomic DNA extracted from the indicated nourseothricin-resistant transformants or from *mac1*Δ as a negative control. M, molecular size markers. **(D, E)** Real-time RT-qPCR analysis was performed in the indicated strains grown in the absence (-Cu) or presence (+Cu) of 100 µM $CuSO_4$. Transcript levels of the indicated genes are expressed relative to those of the wt strain in -Cu conditions. Bars represent standard deviations (n = 3, biological replicates). p-values: ns>0.05, *<0.05, **<0.01 versus *mac1*Δ, under the same condition, according to two-tailed unpaired Student's t test.
