# Supplementary material for: Copper acquisition is essential for plant colonization and virulence in a root-infecting vascular wilt fungus
Source: PLoS Pathog. 2024 Nov 4;20(11):e1012671. doi: 10.1371/journal.ppat.1012671 (PMC11563359; doi:10.1371/journal.ppat.1012671)
Supplement: S10 Fig — (A) Physical map of the Fo-mClover3 expression cassette used to transform mac1Δ or mac1Δctr3OEfre9OE #4 strains. Relative positions of the primers used for diagnostic PCR are indicated. (B, C) Agarose gel electrophoresis of PCR products obtained using the primer pair Gpda4 and 3XFLAGrev with genomic DNA extracted from the indicated phleomycin-resistant transformants or mac1Δ or mac1Δctr3OEfre9OE #4 as negative controls. M, molecular size markers. (PDF) [file ppat.1012671.s010.pdf]

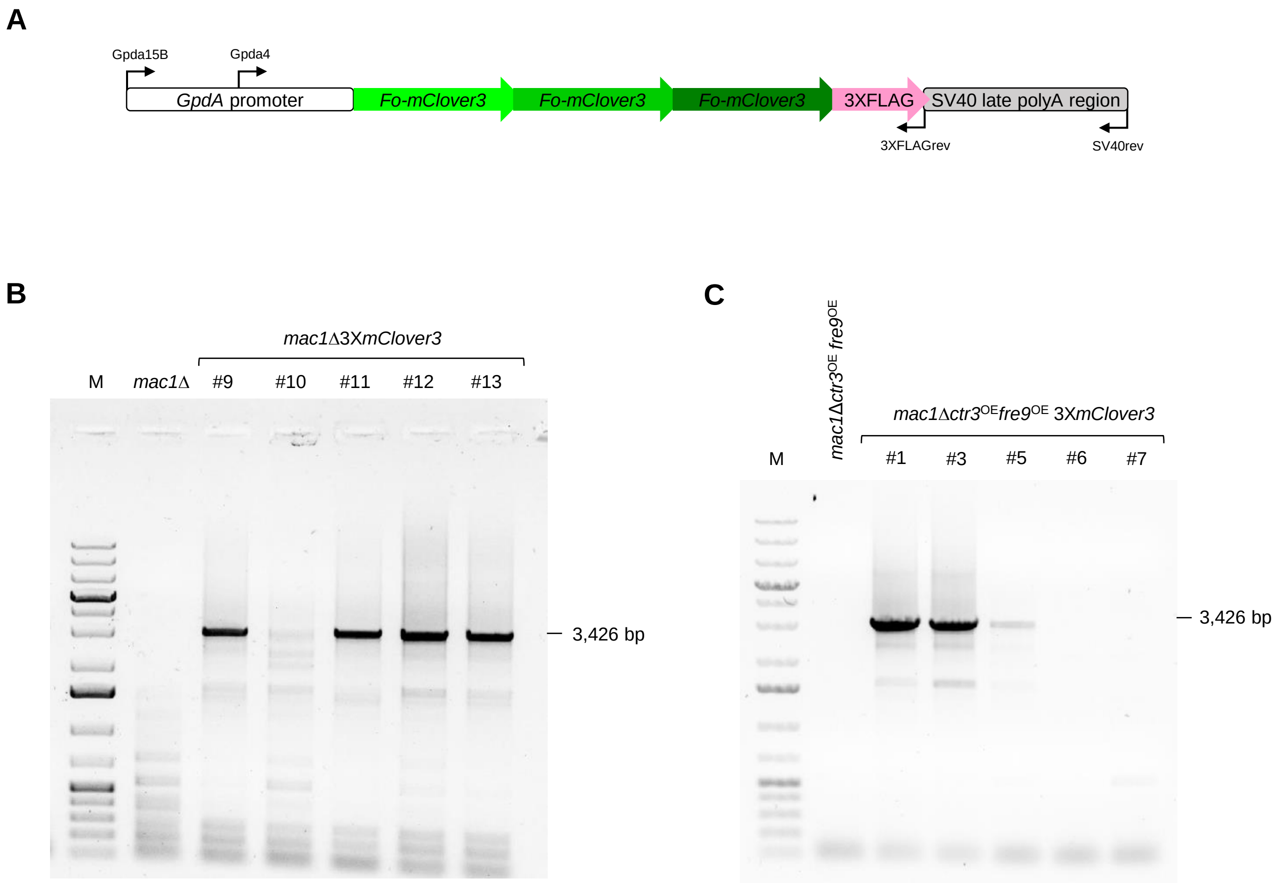

S10 Fig. Generation of fluorescent *mac1*Δ- and *mac1*Δ*ctr3*^{OE}*fre9*^{OE} strains expressing 3X*mClover3*. (A) Physical map of the *Fo-mClover3* expression cassette used to transform *mac1*Δ or *mac1*Δ*ctr3*^{OE}*fre9*^{OE} #4 strains. Relative positions of the primers used for diagnostic PCR are indicated. **(B, C)** Agarose gel electrophoresis of PCR products obtained using the primer pair Gpda4 and 3XFLAGrev with genomic DNA extracted from the indicated phleomycin-resistant transformants or *mac1*Δ or *mac1*Δ*ctr3*^{OE}*fre9*^{OE} #4 as negative controls. M, molecular size markers.
