# Supplementary material for: Copper acquisition is essential for plant colonization and virulence in a root-infecting vascular wilt fungus
Source: PLoS Pathog. 2024 Nov 4;20(11):e1012671. doi: 10.1371/journal.ppat.1012671 (PMC11563359; doi:10.1371/journal.ppat.1012671)
Supplement: S11 Fig — Tomato root colonization of the indicated F. oxysporum strains expressing 3XFo-mClover3 at 4 dpi. Fungal fluorescence (mClover3, green) is overlaid with propidium iodide staining of the plant cell wall (PI, magenta). The two images were merged using ImageJ v1.8. The images shown are representative of at least three lateral secondary roots from six different tomato plants. Scale bar, 50 μm. (PDF) [file ppat.1012671.s011.pdf]

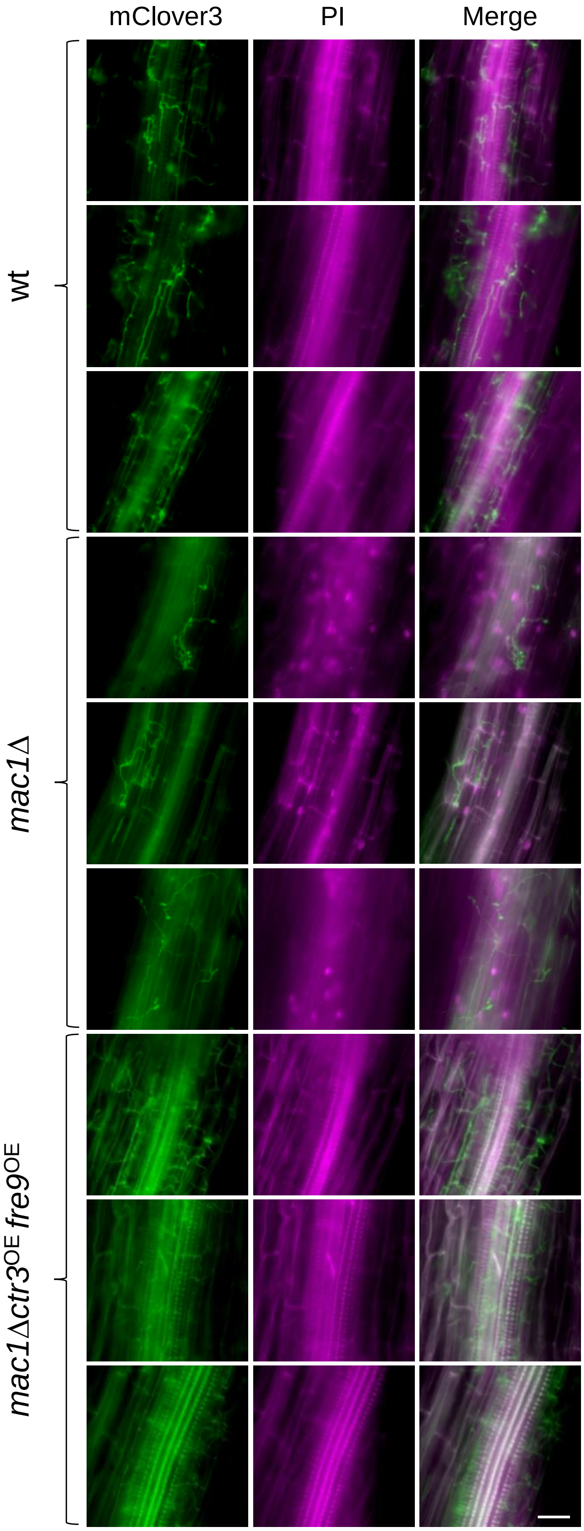

S11 Fig. Overexpression of the high affinity copper transporter *ctr3* and the metalloreductase *fre9* rescues vascular colonization of tomato roots in the *mac1*Δ mutant. Tomato root colonization of the indicated *F. oxysporum* strains expressing 3X*Fo-mClover3* at 4 dpi. Fungal fluorescence (mClover3, green) is overlaid with propidium iodide staining of the plant cell wall (PI, magenta). The two images were merged using ImageJ v1.8. The images shown are representative of at least three lateral secondary roots from six different tomato plants. Scale bar, 50 µm.
