# Supplementary material for: Copper acquisition is essential for plant colonization and virulence in a root-infecting vascular wilt fungus
Source: PLoS Pathog. 2024 Nov 4;20(11):e1012671. doi: 10.1371/journal.ppat.1012671 (PMC11563359; doi:10.1371/journal.ppat.1012671)
Supplement: S12 Fig — (A, B) Kaplan-Meier plot showing survival of groups of 15 G. mellonella larvae after injection into the hemocoel of 1.6x10^5^ microconidia of the indicated F. oxysporum strains or phosphate-buffered saline (PBS) as a negative control. Insects were maintained at 30°C. p-value: ****<0.0001 versus the wt according to Log-rank (Mantel-Cox) test. Data shown are from one representative experiment. Experiments were performed at least two times with similar results. (PDF) [file ppat.1012671.s012.pdf]

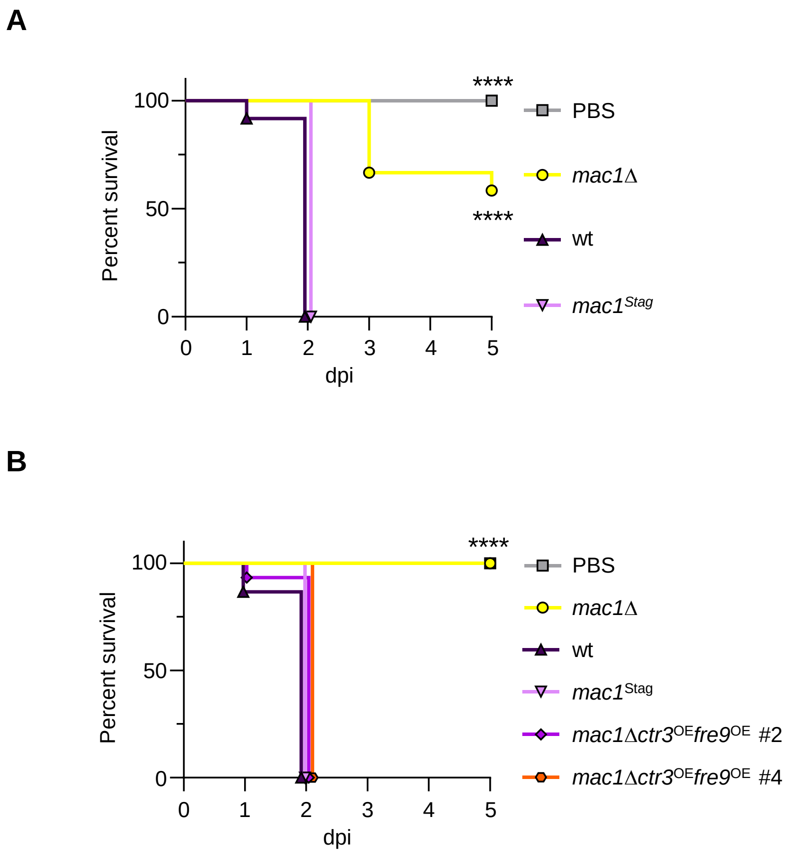

S12 Fig. Overexpression of the high affinity copper transporter *ctr3* and the metalloreductase *fre9* rescues restores virulence of the *mac1*Δ mutant on the animal host *Galleria mellonella*. (A, B) Kaplan-Meier plot showing survival of groups of 15 *G. mellonella* larvae after injection into the hemocoel of $1.6x10^5$ microconidia of the indicated *F. oxysporum* strains or phosphate-buffered saline (PBS) as a negative control. Insects were maintained at 30 ºC. *p*-value: ****<0.0001 versus the wt according to Log-rank (Mantel-Cox) test. Data shown are from one representative experiment. Experiments were performed at least two times with similar results.
