# Supplementary material for: Copper acquisition is essential for plant colonization and virulence in a root-infecting vascular wilt fungus
Source: PLoS Pathog. 2024 Nov 4;20(11):e1012671. doi: 10.1371/journal.ppat.1012671 (PMC11563359; doi:10.1371/journal.ppat.1012671)
Supplement: S13 Fig — Raw gene counts used to evaluate the level of correlation between biological replicates using Pearson’s correlation. Pearson‘s correlation matrix were performed in R (v4.3.0) statistical language and environment, the core function from the stats base package and the corrplot package v0.92 were used for the analysis. (PDF) [file ppat.1012671.s013.pdf]

	R6_DmA3	R6_wt1	R6_wt3	R6_wt2	R6_DmA1	R6_DmA2	R2_wt3	R2_wt1	R2_wt2	R2_DmA1	R2_DmA2	R2_DmA3	DmA_0Cu3	DmA_1Cu3	wt_1Cu2	DmA_0Cu1	DmA_1Cu1	wt_0Cu3	wt_1Cu3	wt_0Cu1	wt_1Cu1	DmA_0Cu2	wt_0Cu2	DmA_1Cu2
R6_DmA3	1	0.99	0.99	0.99	0.99	0.98	0.81	0.8	0.8	0.8	0.8	0.8	0.72	0.72	0.72	0.72	0.73	0.72	0.74	0.74	0.73	0.68	0.71	0.71
R6_wt1	0.99	1	1	0.99	0.98	0.97	0.79	0.78	0.78	0.78	0.79	0.78	0.72	0.72	0.72	0.72	0.72	0.72	0.73	0.73	0.73	0.68	0.7	0.7
R6_wt3	0.99	1	1	0.97	0.97	0.95	0.75	0.75	0.75	0.74	0.75	0.74	0.67	0.67	0.67	0.67	0.68	0.67	0.69	0.68	0.68	0.63	0.66	0.66
R6_wt2	0.99	0.99	0.97	1	0.99	0.99	0.87	0.87	0.87	0.86	0.87	0.86	0.79	0.79	0.79	0.79	0.8	0.79	0.81	0.81	0.81	0.75	0.78	0.78
R6_DmA1	0.99	0.98	0.97	0.99	1	1	0.87	0.86	0.86	0.86	0.86	0.86	0.78	0.79	0.79	0.79	0.79	0.79	0.81	0.8	0.8	0.74	0.77	0.77
R6_DmA2	0.98	0.97	0.95	0.99	1	1	0.89	0.89	0.89	0.89	0.89	0.89	0.81	0.81	0.81	0.81	0.82	0.81	0.83	0.83	0.82	0.76	0.8	0.79
R2_wt3	0.81	0.79	0.75	0.87	0.87	0.89	1	1	1	1	1	1	0.91	0.91	0.91	0.91	0.92	0.9	0.93	0.93	0.93	0.85	0.89	0.89
R2_wt1	0.8	0.78	0.75	0.87	0.86	0.89	1	1	1	1	1	1	0.9	0.9	0.9	0.91	0.91	0.9	0.93	0.93	0.92	0.84	0.88	0.88
R2_wt2	0.8	0.78	0.75	0.87	0.86	0.89	1	1	1	1	1	1	0.9	0.9	0.9	0.9	0.91	0.89	0.93	0.92	0.92	0.84	0.88	0.88
R2_DmA1	0.8	0.78	0.74	0.86	0.86	0.89	1	1	1	1	1	1	0.9	0.91	0.91	0.91	0.92	0.9	0.93	0.93	0.92	0.85	0.89	0.89
R2_DmA2	0.8	0.79	0.75	0.87	0.86	0.89	1	1	1	1	1	1	0.9	0.9	0.9	0.91	0.91	0.9	0.93	0.93	0.92	0.84	0.88	0.88
R2_DmA3	0.8	0.78	0.74	0.86	0.86	0.89	1	1	1	1	1	1	0.91	0.91	0.91	0.91	0.92	0.9	0.93	0.93	0.93	0.85	0.89	0.89
DmA_0Cu3	0.72	0.72	0.67	0.79	0.78	0.81	0.91	0.9	0.9	0.9	0.9	0.91	1	1	1	1	1	0.99	0.99	0.99	1	0.98	0.99	0.99
DmA_1Cu3	0.72	0.72	0.67	0.79	0.79	0.81	0.91	0.9	0.9	0.91	0.9	0.91	1	1	1	1	1	1	1	1	1	0.98	0.99	0.99
wt_1Cu2	0.72	0.72	0.67	0.79	0.79	0.81	0.91	0.9	0.9	0.91	0.9	0.91	1	1	1	1	1	1	0.99	1	1	0.98	0.99	1
DmA_0Cu1	0.72	0.72	0.67	0.79	0.79	0.81	0.91	0.91	0.9	0.91	0.91	0.91	1	1	1	1	1	0.99	0.99	0.99	1	0.98	0.99	1
DmA_1Cu1	0.73	0.72	0.68	0.8	0.79	0.82	0.92	0.91	0.91	0.92	0.91	0.92	1	1	1	1	1	0.99	1	1	1	0.98	0.99	0.99
wt_0Cu3	0.72	0.72	0.67	0.79	0.79	0.81	0.9	0.9	0.89	0.9	0.9	0.9	0.99	1	1	0.99	0.99	1	0.99	1	1	0.98	0.99	0.99
wt_1Cu3	0.74	0.73	0.69	0.81	0.81	0.83	0.93	0.93	0.93	0.93	0.93	0.93	0.99	1	0.99	0.99	1	0.99	1	1	1	0.96	0.98	0.98
wt_0Cu1	0.74	0.73	0.68	0.81	0.8	0.83	0.93	0.93	0.92	0.93	0.93	0.93	0.99	1	1	0.99	1	1	1	1	1	0.97	0.99	0.99
wt_1Cu1	0.73	0.73	0.68	0.81	0.8	0.82	0.93	0.92	0.92	0.92	0.92	0.93	1	1	1	1	1	1	1	1	1	0.97	0.99	0.99
DmA_0Cu2	0.68	0.68	0.63	0.75	0.74	0.76	0.85	0.84	0.84	0.85	0.84	0.85	0.98	0.98	0.98	0.98	0.98	0.98	0.96	0.97	0.97	1	0.99	0.99
wt_0Cu2	0.71	0.7	0.66	0.78	0.77	0.8	0.89	0.88	0.88	0.89	0.88	0.89	0.99	0.99	0.99	0.99	0.99	0.99	0.98	0.99	0.99	0.99	1	0.99
DmA_1Cu2	0.71	0.7	0.66	0.78	0.77	0.79	0.89	0.88	0.88	0.89	0.88	0.89	0.99	0.99	1	1	0.99	0.99	0.98	0.99	0.99	0.99	0.99	1

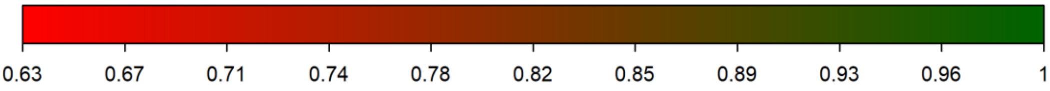

S13 Fig. Pearson’s correlation matrix of *Fol4287* transcriptomes. Raw gene counts used to evaluate the level of correlation between biological replicates using Pearson’s correlation. Pearson‘s correlation matrix were performed in R (v4.3.0) statistical language and environment, the core function from the stats base package and the corrplot package v0.92 were used for the analysis.
