# Supplementary material for: Copper acquisition is essential for plant colonization and virulence in a root-infecting vascular wilt fungus
Source: PLoS Pathog. 2024 Nov 4;20(11):e1012671. doi: 10.1371/journal.ppat.1012671 (PMC11563359; doi:10.1371/journal.ppat.1012671)
Supplement: S14 Fig — Transformed raw counts (vst function from DESeq2 R package) per gene were used as variable for prcomp function from stats R package. PCA shows the samples in the 2D plane spanned by their first two principal components. Sample color is coded according to the experimental condition. (PDF) [file ppat.1012671.s014.pdf]

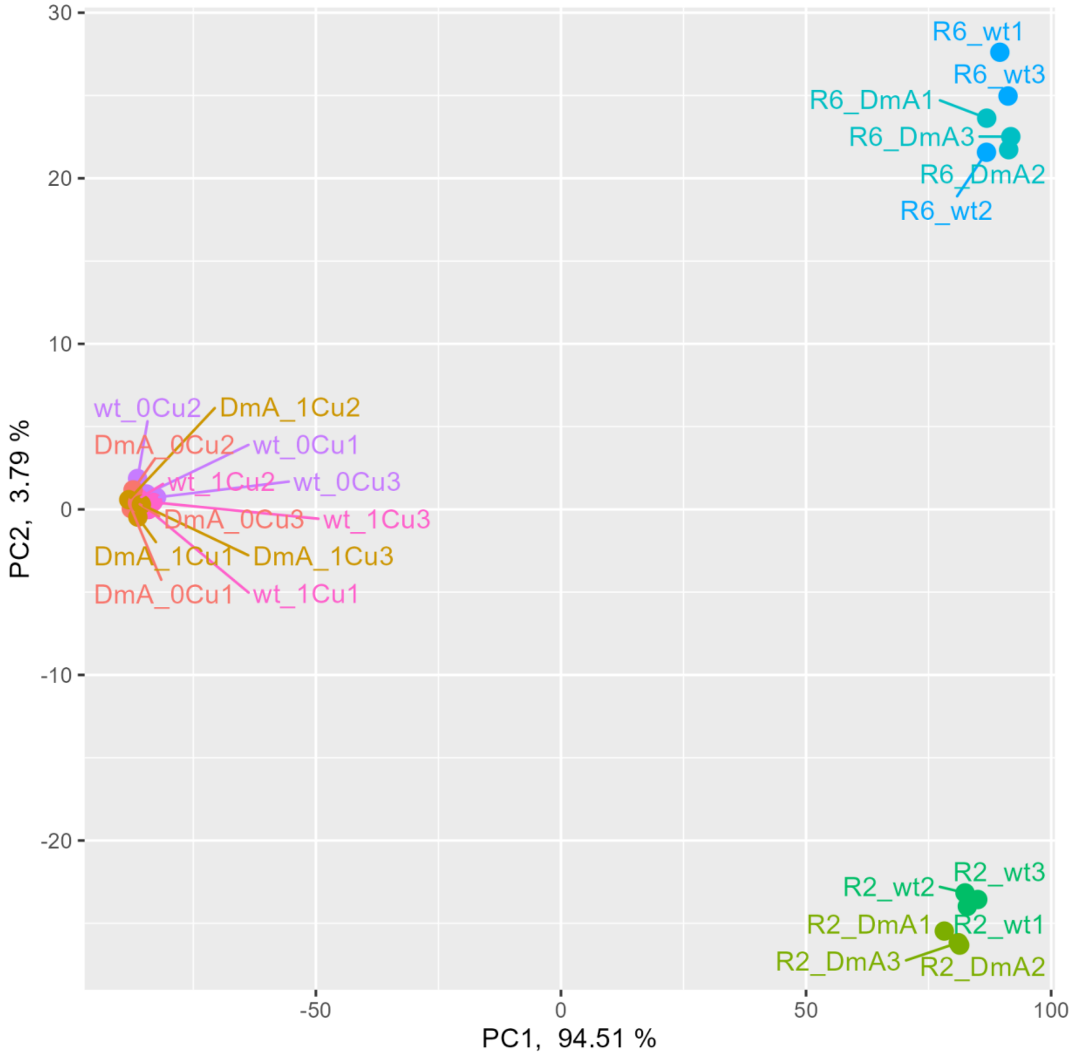

S14 Fig. Principal Component Analysis (PCA) of the RNA-seq data demonstrating the reproducibility of the three biological replicates. Transformed raw counts (vst function from DESeq2 R package) per gene were used as variable for prcomp function from stats R package. PCA shows the samples in the 2D plane spanned by their first two principal components. Sample color is coded according to the experimental condition.
