# Supplementary material for: Copper acquisition is essential for plant colonization and virulence in a root-infecting vascular wilt fungus
Source: PLoS Pathog. 2024 Nov 4;20(11):e1012671. doi: 10.1371/journal.ppat.1012671 (PMC11563359; doi:10.1371/journal.ppat.1012671)
Supplement: S1 Table — (PDF) [file ppat.1012671.s015.pdf]

S1 Table. Fungal strains and tomato cultivars used in this study.

Species	Strain	Features	Reference
Fusarium oxysporum* f. sp. *lycopersici	*Fol4287*	wild-type (f. sp. *lycopersici*, Race 2)	FGSC 9935
	Fol4287 3X*mClover3*	Insertion of 3X-*mClover3* in *Fol4287*. Hyg^R	[1]
	*mac1*Δ	Deletion of the transcription factor *mac1*. Hyg^R	This study
	mac1^{Stag}	Complementation of *mac1*Δ with the *Fol4287* *mac1* allele fused to the Stag epitope	This study
	Mac1^{clover}	Complementation of *mac1*Δ with the *Fol4287* *mac1* allele fused to 1X-*mClover3* under control of the *gpdA* promoter of *A. nidulans*. $Phleo^R$	This study
	*mac1*Δ3X*mClover3*	Ectopic integration of 3X-*mClover3* into *mac1*Δ. $Phleo^R$	This study
	*mac1*Δ*ctr3*^{OE}	Ectopic integration of the *Fol4287 ctr3* gene under control of the *gpdA* promoter of *A. nidulans* into *mac1*Δ. Nat^R	This study
	*mac1*Δ*ctr3*^{OE} *fre9*^{OE}	Ectopic integrations of the *Fol4287 ctr3* and *fre9* genes under control of the *gpdA* promoter of *A. nidulans* into *mac1*Δ. Nat^R	This study
	*mac1*Δ*ctr3*^{OE} *fre9*^{OE} 3X*mClover3*	Insertion of 3X-*mClover3* in *mac1*Δ*ctr3*^{OE}*fre9*^{OE}. $Phleo^R$	This study
	*ctr1a*Δ	Deletion of the Ctr copper transporter *ctr1a*. Neo^R	This study
	*ctr3*Δ	Deletion of the Ctr copper transporter *ctr3*. Hyg^R	This study
	*ctr3*Δ*ctr1a*Δ	Deletion of the Ctr copper transporters *ctr3* and *ctr1a*. Hyg^R/Neo^R	This study

References

1. Redkar A, Sabale M, Schudoma C, Zechmann B, Gupta YK, et al. (2022) Conserved secreted effectors contribute to endophytic growth and multihost plant compatibility in a vascular wilt fungus. Plant Cell 34: 3214-3232.
