# Supplementary material for: Copper acquisition is essential for plant colonization and virulence in a root-infecting vascular wilt fungus
Source: PLoS Pathog. 2024 Nov 4;20(11):e1012671. doi: 10.1371/journal.ppat.1012671 (PMC11563359; doi:10.1371/journal.ppat.1012671)
Supplement: S2 Table — Lowercase nucleotides do not belong to the original sequence and were introduced to generate overlapping ends for fusion PCR reactions. (PDF) [file ppat.1012671.s016.pdf]

S2 Table. List of primers used in this study. Lowercase nucleotides do not belong to the original sequence and were introduced to generate overlapping ends for fusion PCR reactions.

Gene/Vector	Name	Tm	Sequence
hyg	HygG	62 ºC	CGTTGCAAGACCTGCCTGAA
	HygY	64 ºC	GGATGCCTCCGCTCGAAGTA
phleo	FLE-5	64 ºC	CGGAGCGGTCGAGTTCTGG
	LEO	64 ºC	GCCACGAAGTGCACGCAGTT
neo	NeoG	62 ºC	TGCCCTGAATGAACTGCAAGA
	NeoY	62 ºC	CCAAGTTCTTCAGCAATATCAC
**pAN7-1, pAN8-1, pGEMT-Neo^R**	GpdA15B	62 ºC	CGAGACCTAATACAGCCCCT
	GpdA15nest	62 ºC	ACTCAAATCGACTTCAGCAACA
	GpdA3	60 ºC	GTTGACCTAGCTGATTCTGG
	GpdA4	62 ºC	GAAGTGGAAAGGCTGGTGTG
	Gpda9	62 ºC	GGTGATGTCTGCTCAAGCGG
	TrpC8B	62 ºC	AAACAAGTGTACCTGTGCATTC
**pDNat**	M13-F	78 ºC	CGCCAGGGTTTTCCCAGTCACGAC
	M13-R	68 ºC	AGCGGATAACAATTTCACACAGGA
**pUC57- 3X*Fom Clover3*-3XFLAG**	Linker-ruby-for	60 ºC	cggcggttctcatATGGTCTCTAAGGGCGAGG
	EYFP-Rev	64 ºC	TTACTTGTACAGCTCGTCCATG
	3XFLAGrev	62 ºC	TTAAAGCTTATCGTCATCGTCC
	SV40-rev-nest	62 ºC	CTAGATACCACATTTGTAGAGG
	SV40-rev	62 ºC	CTTTTGCTGGCCTTTTGCTCA
**actin**	Act-q7	62 ºC	ATGTCACCACCTTCAACTCCA
	Act-q8	62 ºC	CTCTCGTCGTACTCCTGCTT
mac1	Mac1-5'-F	60 ºC	GCTTTATGGTGGTGTTGGTG
	Mac1-5'-Fn	60 ºC	ATGGAAACTTGCTCACTCGG
	Mac1-5'-R	62 ºC	GATGACAGAGGTGGAGCAAG
	Mac1-hph-F	64 ºC	atccgtcttgctccacctctgtcatcCGAGACCTAATACAGCCCCTA
	Mac1-hph-R	64 ºC	ccaatggcgagtttgaaaggttgatCCTGTGCATTCTGGGTAAACG
	Mac1-3'-F	62 ºC	ATCAACCTTTCAAACTCGCCC
	Mac1-3'-Rn	62 ºC	CCATAGTCATCTTCATCGTCC
	Mac1-3'-R	62 ºC	CTGAAGTGTGGGAATGCCTG
	Mac1-ORF-STOP-R	60 ºC	GGCCTGGCCTTCTGCTTC
	Mac1-Stag-F	62 ºC	cagcggtggaagcagaaggccaggccGGAGCTGGTGCAGGCGCT
	Mac1-Stag-R	62 ºC	agctctcatactctcagaggtgttttTTAGCTGTCCATGTGCTGGC
	Mac1-TER-F	62 ºC	AAAACACCTCTGAGAGTATGAG
	GpdA-Mac1-R	60 ºC	atttttattccattgatgagaggcatGGAAAAGAAAGAGAAAAGAAAAG
	Mac1-ATG-F	58 ºC	ATGCCTCTCATCAATGGAATA
	Mac1-Clover-F	60 ºC	cagcggtggaagcagaaggccaggccATGGTCTCTAAGGGTGAGGA
	Clover-Mac1-Ter-R	58 ºC	agctctcatactctcagaggtgtttttaCTTGTAAAGCTCATCCATGC
	Mac1-qPCR-F	60 ºC	TGATGATGTCGCCTTTTCCG
	Mac1-qPCR-R	60 ºC	TACTTGTCTCCATTGCTCCG
ctr1a	Ctr1a-5'-F	62 ºC	GCTCCATAAATGATCGGTATT
	Ctr1a-5'-Fn	62 ºC	AAGCACAACAGCCTCAAGTC
	Ctr1a-5'-R	62 ºC	TAACGGAGGGACGGTTCGC
	Ctr1a-3'-F	62 ºC	TTCGGCAGCAAATGATATTAG
	Ctr1a-3'-Rn	62 ºC	CTGAGTTTGCCTTCCCGAG
	Ctr1a-3'-R	62 ºC	TGTTGCGTGTTTGTGCCTG

Gene/Vector	Name	Tm	Sequence
ctr3	Ctr3-5’-F	62 ºC	ATGTCGTAGAATAGCCCAAAG
	Ctr3-5’-Fn	60 ºC	GAAGACAGCGAGGATAGTAG
	Ctr3-5'-R	62 ºC	GATGAGTTGTTAGGTTGGTATT
	Ctr3-3’-F	62 ºC	ATACGATGGAGGTTTTACATGA
	Ctr3-3'-Rn	60 ºC	AAGCCACCATTCTCGTCTCA
	Ctr3-3'-R	62 ºC	CTCAGGTCATAAGTTGGTTGG
	Ctr3-FOXG_07770-F	62 ºC	GCGTTATCCTCCTCGTTATTC
	Ctr3-FOXG_07770-R	60 ºC	GTGAAGCTCGATATGTAAGTG
	Gpda-Ctr3	62 ºC	cccgcttgagcagacatcaccATGGATGGAATGGGCGGTAG
fre9	Gpda-Fre9	62 ºC	cccgcttgagcagacatcaccaTGGTGTCTCTCAGCCAGCT
	Fre9-3’-Rn	62 ºC	TGGCGAGAATGATGACTTCCT
	Fre9-3’-R	62 ºC	CTGGGGTTGAAGTAGGAGTG
	Fre9-qPCR-F	62 ºC	CCGTCTATACCAGCCTCAAC
	Fre9-qPCR-R	62 ºC	AGGAGTGATGGAAGCAAGAAG
