# Supplementary material for: Copper acquisition is essential for plant colonization and virulence in a root-infecting vascular wilt fungus
Source: PLoS Pathog. 2024 Nov 4;20(11):e1012671. doi: 10.1371/journal.ppat.1012671 (PMC11563359; doi:10.1371/journal.ppat.1012671)
Supplement: S3 Table — (PDF) [file ppat.1012671.s017.pdf]

S3 Table. Plasmids used in this study.

Plasmid	Features	Reference
**pAN7-1**	Derived from pUC18. Carrier of the phosphotransferase hygromycin B (*hph*) gene from *Streptomyces spp*., under the control of the *gpdA* promoter and the *trpC* terminator of *A. nidulans*	[1]
**pAN8-1**	Carrier of the phleomycin resistance gene under control of the *gpdA* promoter and the *trpC* terminator of *A. nidulans* cloned in pUC18	[2]
**pDNat**	Carrier of the *nat1* gene under the control of the *trpC* promoter of *A. nidulans*	[3]
**pGEMT-Neo**	Carrier of the neomycin resistance gene under control of the *gpdA* promoter and the *trpC* terminator of *A. nidulans*	[4]
**pUC57-3X*FomClover*3**	Derived from pUC57; *A. nidulans gpdA* promoter; 3 copies of a *F. oxysporum* codon optimized *mClover3* (*Fo-mClover3*); 3 copies of the FLAG octapeptide tag coding region (3XFLAG).	[5]
**pUC57-1X*FomClover*3**	Derived from pUC57- 3X*Fo-mClover*- 3XFLAG. Two of the three copies of FomClover3 were removed.	This study
S-tag::pyrG	Carrier of a 4XGA-Stag epitope with the *pyrG* allele of *A. fumigatus*	[6]

References

1. Punt PJ, Oliver RP, Dingemanse MA, Pouwels PH, van den Hondel CA (1987) Transformation of *Aspergillus* based on the hygromycin B resistance marker from *Escherichia coli*. Gene 56: 117-124.
2. Mattern IE, Punt PJ, Van den Hondel CA (1988) A vector for *Aspergillus* transformation conferring phleomycin resistance. Fungal Genetics Reports 35.
3. Kopke K, Hoff B, Kuck U (2010) Application of the *Saccharomyces cerevisiae* FLP/FRT recombination system in filamentous fungi for marker recycling and construction of knockout strains devoid of heterologous genes. Appl Environ Microbiol 76: 4664-4674.
4. Fernandes TR, Segorbe D, Prusky D, Di Pietro A (2017) How alkalinization drives fungal pathogenicity. PLoS Pathog 13: e1006621.
5. Redkar A, Sabale M, Schudoma C, Zechmann B, Gupta YK, et al. (2022) Conserved secreted effectors contribute to endophytic growth and multihost plant compatibility in a vascular wilt fungus. Plant Cell 34: 3214-3232.
6. Pinar M, Arst HN, Jr., Pantazopoulou A, Tagua VG, de los Rios V, et al. (2015) TRAPPII regulates exocytic Golgi exit by mediating nucleotide exchange on the Ypt31 ortholog RabERAB11. Proc Natl Acad Sci U S A 112: 4346-4351.
